# Changing eating habits: what does it really mean for patients who do not complain about eating difficulties?

**DOI:** 10.1590/S1516-31802004000500001

**Published:** 2004-09-01

Alimentation is necessary for survival. The ability to eat depends, among other factors, on the capacity to swallow. This is a physiologically complex process that is subject to the integrity of the system through which it is executed at various levels.^1-3^ The process can be altered by illness or medical and surgical interventions.^4^ On the other hand, clinical treatments that can aid swallowing are today available.^5-7^ However, information on the consequences of illness or interventions on the swallowing process, or guidance on eating habits, is not always available.

In this edition of the São Paulo Medical Journal, Pillon et al.^8^ evaluate the alterations in the eating habits of 36 patients submitted to frontolateral partial and total laryngectomy and conclude that difficulties in swallowing are frequent. The strength of the article lies in its study of swallowing, a field that demands multidisciplinary contributions.^9^ Recently, dysphagia and its consequences have been researched by different professionals with the aim of improving the therapeutic process and quality of life for those who suffer swallowing difficulties.^10,11^ Besides evaluating a matter that is not always emphasized in clinical practice, the study by Pillon et al.^8^ brings out other important questions regarding eating habits. A very interesting finding was that, even among the patients who were not initially diagnosed as dysphagic, 13 (36%) exhibited some feeding difficulty. Sixteen (44%) needed to alter the consistency of their food and make other adaptations in order to be able to eat. Difficulties were widespread among the patients submitted to total laryngectomy. The observation that patients developed adaptations to facilitate their eating without spontaneously reporting these to their physicians is noteworthy.

After these individuals had undergone surgical interventions on the structures that contribute towards swallowing, they became adapted to their new situation and developed a capability for functional swallowing. They had not experienced any weight loss. The individuals studied did not spontaneously mention the maneuvers they utilized: the additional efforts needed for swallowing and the changes made in the selection and consistency of their food. Nevertheless, it can be presumed that these adaptations had their costs, in social and wellbeing terms.

The study demonstrates that patients should routinely and specifically be asked whether they are having difficulties in eating. The questionnaire devised by the speech therapists contributed towards patients perceiving and being able to name the processes involved in the adaptations that they knowingly had undertaken. Additionally, Pillon et al.^8^ outline a discussion about the breadth of treatment, including the perception of wellbeing in relation to voice deprivation, survival conditions and greater quality of life.

Patients who do not complain about their eating difficul-ties may imagine that it is impossible to overcome them. It is befitting that professionals who are responsible for patient care, following interventions that alter the swallowing mechanisms, should include data on eating habits among the indicators for such patients’ evolution. The success of the treatment can greatly depend on these initiatives.
